# Obstructive Sleep Apnea Monocytes Exhibit High Levels of Vascular Endothelial Growth Factor Secretion, Augmenting Tumor Progression

**DOI:** 10.1155/2018/7373921

**Published:** 2018-05-28

**Authors:** Carolina Cubillos-Zapata, Enrique Hernández-Jiménez, José Avendaño-Ortiz, Victor Toledano, Anibal Varela-Serrano, Isabel Fernández-Navarro, Raquel Casitas, Carlos Carpio, Luis A. Aguirre, Francisco García-Río, Eduardo López-Collazo

**Affiliations:** ^1^The Innate Immune Response Group, Tumor Immunology Lab, IdiPAZ, La Paz University Hospital, Madrid, Spain; ^2^Center for Biomedical Research Network of Respiratory Diseases (CIBERES), Madrid, Spain; ^3^Respiratory Diseases Group, IdiPAZ and Respiratory Service of La Paz University Hospital, Madrid, Spain

## Abstract

Obstructive sleep apnea (OSA) is a syndrome characterized by repeated pauses in breathing induced by a partial or complete collapse of the upper airways during sleep. Intermittent hypoxia (IH), a hallmark characteristic of OSA, has been proposed to be a major determinant of cancer development, and patients with OSA are at a higher risk of tumors. Both OSA and healthy monocytes have been found to show enhanced HIF1*α* expression under IH. Moreover, these cells under IH polarize toward a tumor-promoting phenotype in a HIF1*α*-dependent manner and influence tumor growth via vascular endothelial growth factor (VEGF). Monocytes from patients with OSA increased the tumor-induced microenvironment and exhibited an impaired cytotoxicity in a 3D tumor *in vitro* model as a result of the increased HIF1*α* secretion. Adequate oxygen restoration both *in vivo* (under continuous positive airway pressure treatment, CPAP) and *in vitro* leads the monocytes to revert the tumor-promoting phenotype, demonstrating the plasticity of the innate immune system and the oxygen recovery relevance in this context.

## 1. Introduction

Obstructive sleep apnea (OSA) is a highly prevalent disorder characterized by intermittent episodes of partial or complete obstruction of the upper airway during sleep. OSA is associated with continued intermittent hypoxia (IH), increased inspiratory efforts, and sleep disruption [1]. Experimental and clinical data suggest that cancer is associated with OSA [2–6]; however, the relevance of the innate immune system in this context remains undetermined.

The immune system defends the body against pathogens and tumor progression. Monocytes are crucial to driving potent anticancer responses at early tumor stages. We have previously reported that hypoxia-inducible factor 1-*α* (HIF1*α*) regulates the expression of several proinflammatory negative regulators and antimicrobial genes and influences tissue remodeling in human monocytes [7]. This immunosuppressive role of HIF1*α* is also supported by tumor studies, in which this factor was crucial to polarizing tumor-associated macrophages toward a tumor-promoting phenotype [8, 9].

HIF1*α* also targets several downstream genes, including vascular endothelial growth factor (VEGF) [10]. This molecule induces a number of angiogenic factors and supports tumor progression. The phenomenon has been confirmed in both *in vitro* and *in vivo* intermittent hypoxia (IH) models [5, 6]. Angiogenesis within the primary tumor provides the necessary routes for dissemination, and VEGF-induced changes in vascular permeability promote intra- and extravasation then supporting tumor progression. VEGF has also been well described as a potent inductor of tumor cell growth in various models [11–13].

Both hypoxia and IH enhance HIF1*α* expression, promoting several steps of the metastatic cascade, selecting tumor cell populations and enriching the tumor microenvironment of the primary tumor [14]. Indeed, the factors produced in the tumor microenvironment increase the abilities of tumor cells to grow and escape from immunosurveillance, contributing to tumor progression. In this regard, VEGF is considered one of the main factors; thus, preclinical models and early studies of these approaches have giving promising results [15].

To understand the potential risk of cancer in patients with OSA, we studied the involvement of the innate immune system in this context. Herein, we report that monocytes under IH, either from patients with OSA or from an IH *in vitro* model, produce VEGF in an HIF1*α*-dependent manner and subsequently induce enhanced tumor progression, as validated in a 3D *in vitro* tumor model.

## 2. Methods

### 2.1. Study Design

Patients with OSA were prospectively recruited from the sleep unit of La Paz University Hospital-Cantoblanco, Madrid, Spain. Patients aged between 40 and 65 years with an apnea-hypopnea index of 30 or greater were included in the study.

The diagnosis of OSA was established by respiratory polygraphy (Embletta Gold, ResMed), which included continuous recording of oronasal flow and pressure, heart rate, thoracic and abdominal respiratory movements, and oxygen saturation (SpO_2_). Those tests in which the patients claimed to sleep less than 4 hours or in which there were less than 5 hours of nocturnal recording were repeated.

Exclusion criteria were the following: previous or current treatment with oxygen or mechanical ventilation; underweight (body mass index [BMI] < 18.5 kg/m^2^) or morbid obesity (BMI > 40 kg/m^2^); history of respiratory or cardiovascular disease, including chronic obstructive pulmonary disease, asthma, respiratory failure, hypertension, heart failure, and coronary artery disease; any infectious disease in the previous 3 months; diagnosis of malignancies or chronic inflammatory diseases; and the use of inhaled or systemic corticosteroids or other anti-inflammatory drugs.

Two OSA groups were selected according to the use of continuous positive airway pressure (CPAP): CPAP-naïve patients (the untreated group, OSA) and patients who had been treated with CPAP for at least 6 months (the treated group, CPAP), with a mean daily use of more than 4.5 h per day, as measured with a run-time counter. In the CPAP group, optimal CPAP pressure had been titrated by an auto-CPAP device (REMstar Pro M Series with C-Flex, Philips Respironics) and verified by repeated respiratory polygraphy at the time of inclusion in the study. As a control group, healthy volunteers were selected who were homogeneous in sex, age, smoking habit, and BMI. None of these volunteers were being treated with any type of medication, and the diagnosis of OSA was ruled out by respiratory polygraphy.

La Paz University Hospital's Ethics Committee approved the study (PI-1857), and informed consent was obtained from all the participants.

### 2.2. Characteristics of the Participants

Forty patients with severe OSA were included. Of these, 20 were untreated patients (OSA group) and 20 were patients treated with CPAP for more than 6 months (CPAP group) as a counterpart internal control. In addition, 20 healthy volunteers (HV group) were analyzed.  Table 1 shows the baseline anthropometric and clinical data on these 3 groups. The baseline clinical characteristics were initially similar between the OSA and CPAP groups; however, after 6 months of CPAP treatment, the patients showed recovered clinical values similar to the HV (data not shown).

### 2.3. Reagents and Cell Lines

Dulbecco's modified Eagle's medium (DMEM, Invitrogen) and Roswell Park Memorial Institute 1640 medium (RPMI, Invitrogen) were used for the cell cultures. The VEGF_165_ antibody (R&D Systems) was used for the inhibition assays. All reagents used for cell cultures were tested for endotoxins using the limulus amebocyte lysate test (Lonza). The tumor cell lines (LoVo and BxPC3) were authenticated and free from contamination by bacteria such as mycoplasma. The human VEGF was obtained from Sigma-Aldrich. The intracellular oxygen levels were analyzed by the ability of oxygen to quench the excited state of an oxygen-sensitive probe from intracellular oxygen concentration assay (Abcam, ab197245).

### 2.4. Blood Samples and Peripheral Blood Mononuclear Cells

Blood samples were obtained from the participants between 8:00 and 9:00 AM, following an overnight fast, in order to avoid any circadian cycle effect. Peripheral blood mononuclear cells (PBMCs) were isolated by centrifugation in Ficoll-Histopaque Plus (Amersham Biosciences) for flow cytometry and real-time quantitative polymerase chain reaction (RT-qPCR) analysis. For mechanistic and mRNA expression studies involving monocytes, a specific isolation kit from Miltenyi Biotec was used; cell purity was checked by flow cytometry showing >95% positive cells.

### 2.5. Cell Culture

Normoxic cells were maintained at atmospheric oxygen concentrations (21% O_2_, 5% CO_2_, 37°C). Intermittent hypoxic cells were exposed in the hypoxia chamber (3% O_2_, 5% CO_2_, 37°C) for 12 cycles and changed to preconditioned hypoxic medium (5 minutes) and normoxic medium (10 minutes); the total length of time was 4 hours, as previously described [16]. The use of preconditioned medium was necessary for exposure to hypoxia in an instantaneous manner.

### 2.6. Biomarker Expression by Quantitative Polymerase Chain Reaction

Total RNA was purified from PBMCs and isolated monocytes, using the High Pure RNA isolation kit from Roche Diagnostics. The complementary DNA (cDNA) was obtained by reverse transcription of 1 mg RNA using the High Capacity cDNA Reverse Transcription kit (Applied Biosystems). Gene expression levels were analyzed by qPCR (LightCycler, Roche Diagnostics), using specific primers: HIF1*α* 5′-TTCCAGTTACGTTCCTTCGATCA-3′ (forward) and 5′-TTTGAGGACTTGCGCTTCA-3′ (reverse) and VEGF 5′-GCAGCTTGAGTTAAACGAACG-3′ (forward) and 5′-GCAGCGTGGTTTCTGTATC-3′ (reverse). Relative expression was based on target gene versus housekeeping gene (*β*-actin) levels, and the cDNA copy number of each gene of interest was determined using a 7-point standard curve. The relative expression was calculated using the reference sample in each assay from the standard curve.

### 2.7. Knockdown and Overexpression Studies

The HIF1*α* knockdown was performed by transfecting monocytes with two HIF1*α* Silencer Select small interfering RNAs (siRNAs) and two control siRNAs from Thermo Fisher. HIF1*α* overexpression was obtained by transfecting monocytes with a human HIF1*α* expression plasmid (AM 16708 and 4390824) or a control plasmid (pmaxGFP) from Amaxa Biosystems. Monocytes were nucleoporated using the Amaxa Nucleofector System (Amaxa Biosystems, Lonza).

### 2.8. Cytometric Bead Array

VEGF levels in sera and in the culture supernatants were determined using the VEGF Flex Set (BD Biosciences), following the manufacturer's protocol, and were analyzed by flow cytometry using a BD FACSCalibur flow cytometer (BD Biosciences).

### 2.9. Tumor Spheroid Formation and Cell Viability Assay

For the establishment of the 3D *in vitro* model, we seeded in unison both 500 tumor cells and isolated monocytes per well, at various ratios (1 : 0, 1 : 1, 1 : 2, and 1 : 4) into ultralow attachment 96-well round-bottomed plates. After seeding, the plates were centrifuged 10 minutes at 1500 rpm and incubated in complete DMEM medium for 7 days. This protocol has been previously described [17]. The diameter of the spheroids and cell viability (intracellular acid phosphatase assay) were evaluated at day 7. The tumor cell lines used were BxPc3 (derived from a 61-year-old female patient with a primary adenocarcinoma of the pancreas) and LoVo (isolated from a 56-year-old male patient with an adenocarcinoma of the colon). In addition, we have used tumor cell lines expressing the VEGF receptor [18, 19].

### 2.10. Statistics

Statistical significance was calculated using the Mann–Whitney test and paired *t*-test where appropriate. The correlations were assessed with Spearman's rank correlation for nonnormally distributed data. The differences were considered significant at *p* < .05, and the analyses were conducted using Prism 5.0 software (Graph Pad, USA).

## 3. Results

### 3.1. Monocytes under Intermittent Hypoxia Release VEGF Protein

**Figure 1:**
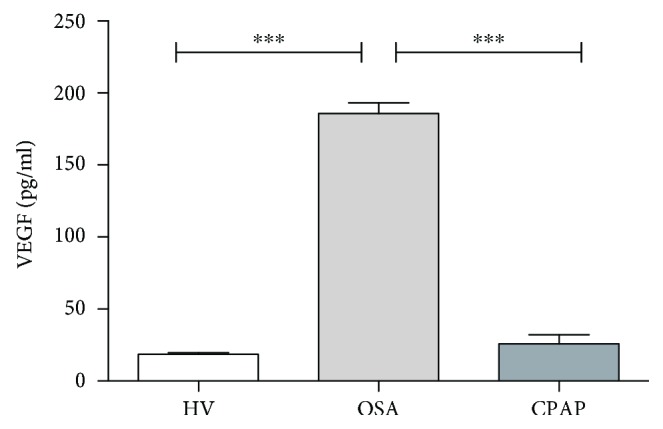
Obstructive sleep apnea monocytes enhance vascular endothelial growth factor secretion. CD14^+^ monocytes were isolated from HV (open bar, *n* = 20 randomly selected), OSA (light gray bar, *n* = 20 randomly selected), and CPAP (dark gray bar, *n* = 20 randomly selected). Monocytes were cultured for 16 hours at 37°C. The VEGF protein levels in the supernatant were analyzed by CBA; ^∗∗∗^ *p* < .0001, using the one-way ANOVA test.

We have previously demonstrated that OSA monocytes exhibit a tumor-associated monocyte (TAM) phenotype [20]. Levels of VEGF in the supernatant from cultures of isolated OSA monocytes were higher than in HV and CPAP (Figure 1). We have also confirmed the increased expression of both HIF1*α* and VEGF mRNA levels in OSA monocytes (Supplementary Figure 1A). Interestingly, HIF1*α* and VEGF mRNA expression from OSA patients indicates a positive correlation with the hypoxia clinical parameter, nighttime oxygen saturation levels < 90% (CT90).

**Figure 2:**
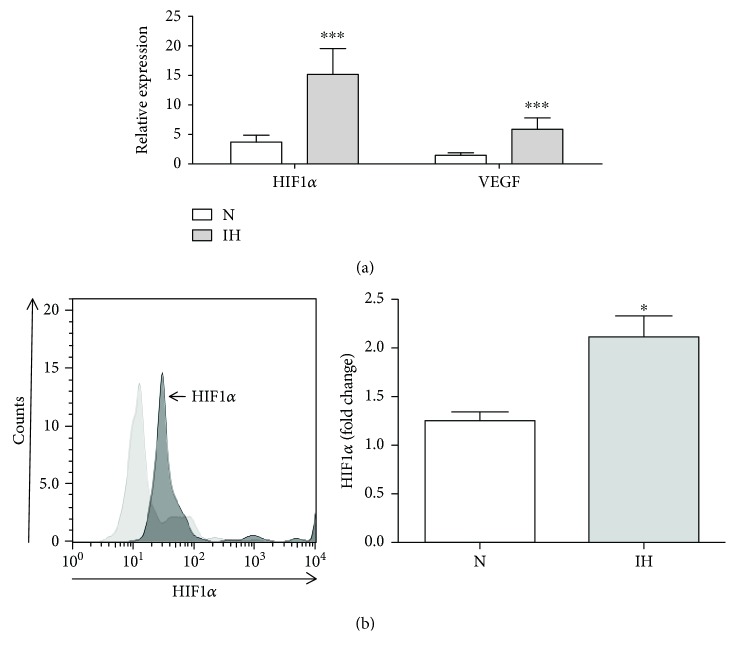
Intermittent hypoxic monocytes enhance vascular endothelial growth factor expression. CD14^+^ monocytes were isolated from HV and cultured under normoxia (N, open bars, *n* = 7) or intermittent hypoxia (IH, light gray bars, *n* = 7) conditions. (a) HIF1*α* and VEGF mRNA expression by qPCR; ^∗∗∗^ *p* < .001, using a paired *t*-test. (b) Intracellular HIF1*α* expression analyzed by flow cytometry (left panel) and fold change of HIF1*α* (right panel); ^∗^ *p* < .05, using a paired *t*-test.

The main characteristic of patients with OSA is IH; therefore, circulating monocytes from patients with OSA were exposed to IH. In an IH *in vitro* model, using cyclic fluctuations from preconditioned hypoxic medium followed by preconditioned normoxic medium, we were able to generate cyclic fluctuations of intracellular oxygen, as previously described by Ryan et al. (Supplementary Figure 2) [16]. The analysis of HIF1*α* and VEGF mRNA levels of isolated monocytes from healthy donors, after IH in an *in vitro* model, corroborated the switch to the tumor-promoting phenotype observed in OSA monocytes, exhibiting high levels of both HIF1*α* and VEGF (Figure 2(a)). The HIF1*α* protein stabilization in monocytes under IH was also corroborated (Figure 2(b)). Altogether, these results indicate that OSA monocytes produce VEGF and could promote a tumorigenic environment.

### 3.2. Monocytes under Intermittent Hypoxia Exhibit VEGF Expression via HIF1*α*

**Figure 3:**
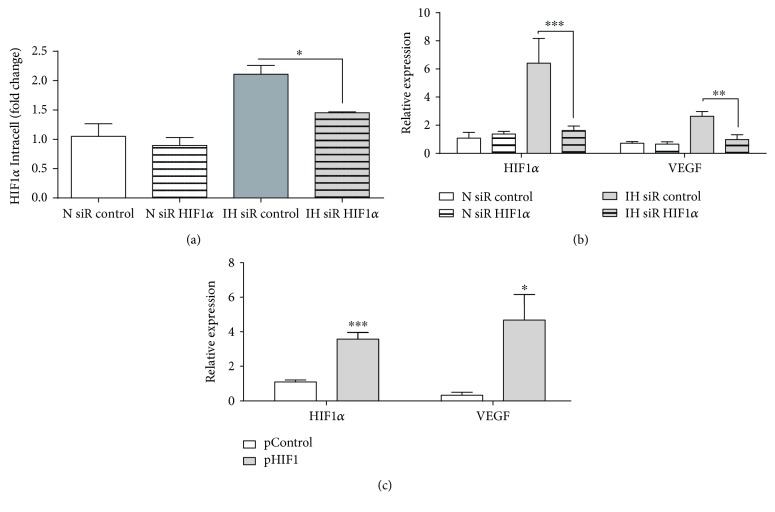
Monocytes exposed to intermittent hypoxia exhibit a VEGF mRNA expression depending on HIF1*α* expression. CD14^+^ monocytes isolated from HV were nucleofected with siRNA control (30 nM, empty bars, *n* = 6) or siRNA HIF1*α* (30 nM, striped bars, *n* = 6). After 16 hours, monocytes were cultured under normoxia (N, open bars, *n* = 6) or IH (light gray bars, *n* = 6) conditions. (a) Intracellular HIF1*α* expression analyzed by flow cytometry; ^∗^ *p* < .05, using a paired *t*-test. (b) HIF1*α* and VEGF mRNA expression by qPCR; ^∗∗^ *p* < .01 and ^∗∗∗^ *p* < .001, using a two-way ANOVA test. (c) CD14^+^ monocytes isolated from HV were nucleofected with control plasmid (0.5 *μ*g, open boxes, *n* = 7) or HIF1*α* plasmid (0.5 *μ*g, light gray boxes, *n* = 7) for 16 hours. HIF1*α* and VEGF mRNA expression by qPCR; ^∗^ *p* < .05 and ^∗∗∗^ *p* < .001, relative to the OSA, using a paired *t*-test.

To evaluate the HIF1*α* role enhancing VEGF production on monocytes under IH, we performed HIF1*α* knockdown assays in healthy monocytes under IH in an *in vitro* model (Figure 3(a)). Our data demonstrated that HIF1*α* knockdown reduced not only the HIF1*α* but also the VEGF production in IH monocytes, suggesting that HIF1*α* plays a crucial role in VEGF induction (Figure 3(b)). Moreover, HIF1*α* overexpression by plasmid transfection in healthy monocytes mimics the resulted phenotype either in patients with OSA or after IH (Figure 3(c)). Altogether, these findings demonstrate the important role of HIF1*α* as a VEGF inducer.

### 3.3. Monocytes from Patients with OSA Exhibit Tumor-Promoting Activity

**Figure 4:**
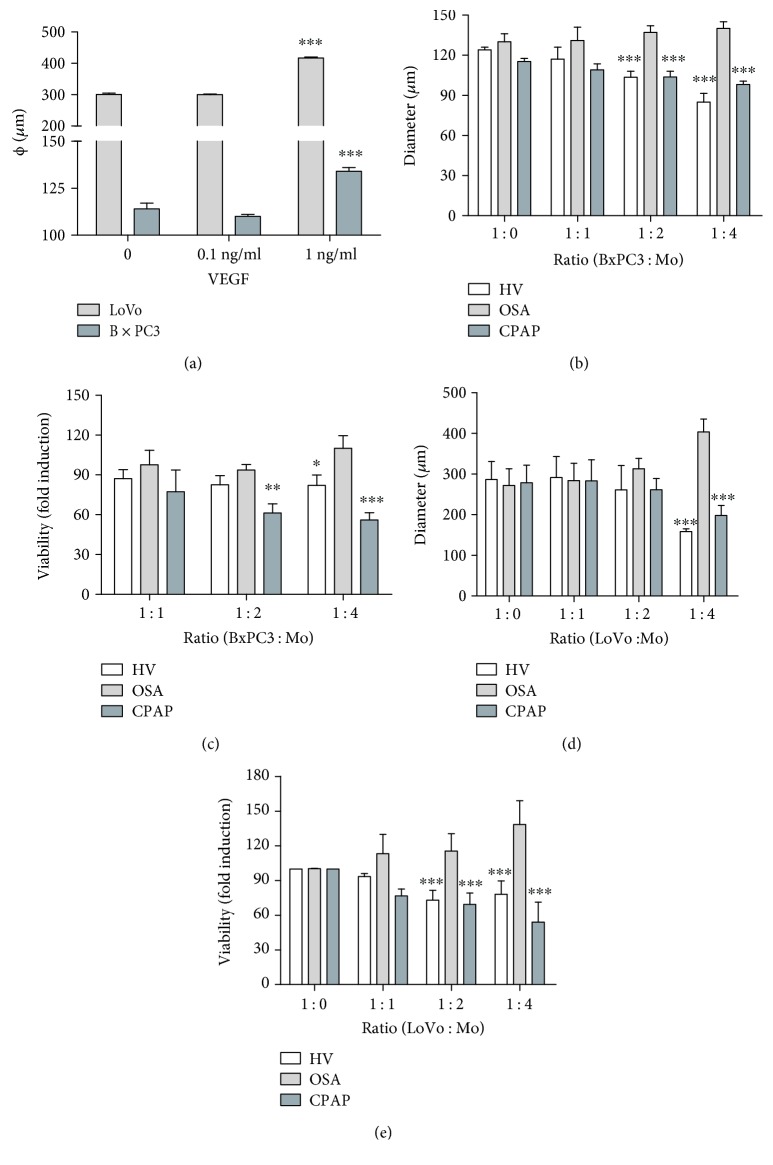
Obstructive sleep apnea monocytes exhibit tumor-promoting activity. CD14^+^ monocytes were isolated from HV (open bars, *n* = 20 randomly selected), OSA (light gray bars, *n* = 20 randomly selected), and CPAP (dark gray bars, *n* = 20 randomly selected). Then, monocytes were cocultured at various ratios in a tumor 3D *in vitro* model using human pancreas (BxPC3) and colon (LoVo) tumor cell lines (3 replicates for each participant). (a) The sizes of the tumor spheres of LoVo and BxPC3 cultured in the presence or not of recombinant human VEGF are shown; ^∗∗∗^ *p* < .001, relative to respective controls, using a one-way ANOVA test. (b) The diameter and (c) viability of BxPC3 are shown. (d) The diameter and (e) viability of LoVo are shown; ^∗^ *p* < .05, ^∗∗^ *p* < .01, and ^∗∗∗^ *p* < .001, relative to respective control ratios, using a one-way ANOVA test.

Furthermore, we evaluated the effect on tumor spheroid size of VEGF protein at various concentrations, using two human cell lines (BxPC3 and LoVo, Figure 4(a)). Next, to study the potential role in tumor progression of VEGF produced by OSA monocytes, we performed a coculture assay with OSA, CPAP, and healthy monocytes at various ratios in an *in vitro* 3D tumor model with the human tumor cell lines from the pancreas (BxPC3) and colon (LoVo) [17]. Tumor sphere sizes were larger in the presence of OSA monocytes compared with CPAP or HV (Figure 4(b)). Accordingly, a significant increase in tumor viability was observed in coculture of BxPC3 with OSA monocytes (Figure 4(c)). Microscopic observation indicated that tumor sphere formation was severely compromised in the presence of CPAP and HV monocytes, but not in cocultures with monocytes from patients with OSA (Supplementary Figure 3). Similar results were obtained for the LoVo cell line (Figures 4(d) and 4(e) and Supplementary Figure 3). In addition, this data also corroborated that OSA monocytes at different ratios decreased the cytotoxic activity compared to CPAP and HV [20].

### 3.4. VEGF Release by OSA Monocytes Enhanced Tumor-Promoting Activity

**Figure 5:**
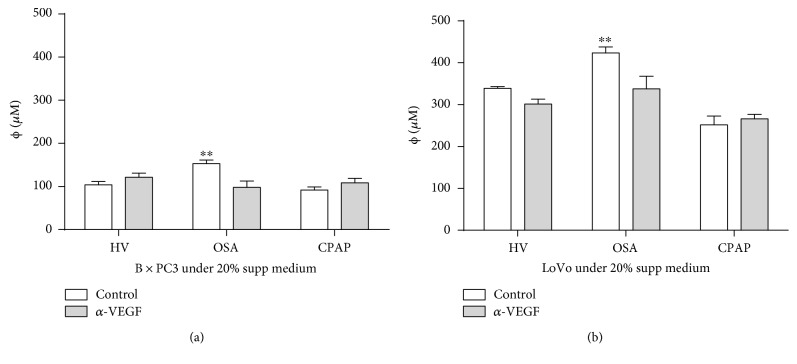
Tumor-promoting activity by obstructive sleep apnea monocytes is mediated by the vascular endothelial growth factor secretion. CD14^+^ monocytes were isolated from HV (open bars, *n* = 20 randomly selected), OSA (light gray bars, *n* = 20 randomly selected), and CPAP (dark gray bars, *n* = 20 randomly selected). Then, monocytes were cocultured at ratio 1 : 2 in a tumor 3D *in vitro* model using human pancreas (BxPC3) or colon (LoVo) tumor cell lines (3 replicates for each participant). Tumor spheroids were cultured in the presence of a blocking VEGF antibody (3 *μ*g/ml, gray bars) or without it (white bars). (a) BxPC3 and (b) LoVo spheroids are shown; ^∗∗^ *p* < .01, using the Mann–Whitney test.

In order to verify the tumor-promoting activity of the VEGF released by OSA monocytes, we performed tumor sphere cultures of both BxPC3 and LoVo, supplemented with 20% of monocyte supernatants from OSA, CPAP, and HV subjects and in the presence or not of a blocking anti-VEGF antibody. In both cases, our data show a significantly reduced size of tumor when the blocking VEGF antibody was added to OSA supplemental medium. This demonstrated the functional role of VEGF protein production in tumor progression. In contrast, the CPAP and HV supplemental medium assays showed no difference between them (Figure 5). Collectively, these data suggest that OSA monocytes are capable of producing VEGF and promoting a tumorigenic environment.

## 4. Discussion

A number of studies indicate that tumor progression is favored under hypoxic conditions [21, 22]. Experimental and clinical data suggest that patients with OSA could be at a higher risk of cancer [2–6, 23]. Several authors using animal models of this pathology have reported that IH during sleep increases tumor growth rates and raises metastatic potential [5, 24]. Some clinical studies have also shown that mortality [2], tumor progression [25], and cancer incidence were greater in patients with severe OSA [3]. Moreover, we have previously described that OSA monocytes exhibited a TAM phenotype that reduces natural killer cell activity [20]. Herein, we report that patients with OSA exhibit a tumor-promoting phenotype on their monocytes, generating a systemic IH environment that compromises antitumor activity of these cells.

In our previous report, we demonstrated that the IH conditions in patients with OSA upregulated HIF1*α* expression. In agreement, our data from functional assays demonstrated that both OSA monocytes and monocytes from IH *in vitro* models exhibited high expression of HIF1*α* and VEGF, together inducing a cancer cell-promoting phenotype, whereas interfering assays reverted this phenotype. Likewise, overexpression of HIF1*α* reproduced the features described in both the IH model and in the patients with OSA. We have previously reported that HIF1*α* played a crucial role in the reprogramming of monocytes toward an alternative phenotype during sepsis [7] and that restoring adequate oxygenation reverts the tumor-promoting phenotype of OSA monocytes, reducing HIF1*α* expression, and subsequently VEGF secretion [20]. Although one limitation of this study resides in the small number of recruited OSA patients, our data corroborates the relation of HIF1*α* mRNA expression with CT90 in this pathology, hence the correlation between VEGF and CT90.

Additionally, tumor 3D spheroids cultured with CPAP monocytes showed an antitumor immune response similar to that of monocytes from healthy donors, which corroborates the crucial role of HIF1*α* and VEGF in OSA monocytes. In accordance with these data, CPAP treatment provides potential clinical relevance to increase the immune response against transformed cells at least on antitumor immune response from monocytes. As far as we know, there has been no clinical evidence that treatment with CPAP reduced the incidence or progression of cancer in patients with OSA; however, several studies that have analyzed the association between OSA and cancer mortality have excluded patients with good adherence to CPAP treatment [2, 26].

It is well accepted that VEGF is necessary for tumor progression and metastasis; in fact, angiogenesis inhibitors are clinically validated anticancer drugs. VEGF is widely considered a main target of the therapeutic treatment for tumor angiogenesis. Indeed, VEGF has been demonstrated to be a tumor inductor, enhancing the growth of tumor cell lines under *in vitro* conditions. Moreover, Du et al. have shown that monocytes derived from bone marrow cells regulate tumor angiogenesis and invasion though HIF1*α* and VEGF production [11]. In the present study, we have demonstrated using our 3D *in vitro* model that an anti-VEGF is able to decrease tumor proliferation in terms of cell viability and tumor-spheroid size in two different tumor cell lines. These results are in line with other studies which largely demonstrate that VEGF serum levels are correlated with apnea hypopanea and oxygen desaturation indexes, besides the fact that VEGF levels decrease after 6 months on CPAP treatment, altogether indicating the association of the severity of the OSA pathology with VEGF serum levels [27–30].

Collectively, our data support the association of patients with OSA and cancer incidence. The IH environment increases HIF1*α*, and subsequently VEGF, in monocytes, switching them to a tumor-promoting phenotype.

## Figures and Tables

**Table 1:** Baseline characteristics of the study participants.

	OSA group (*n* = 20)	CPAP group (*n* = 20)		Healthy volunteers (*n* = 20)	*p* value^¶^
		Before treatment^#^	After treatment		
Age—years	56 ± 11	59 ± 88	—	45 ± 9	.865
Male sex—number (%)	45 (80.3)	50 (81.2)	—	40 (84.5)	.108
Body mass index—kg/m^2^	32.3 ± 5.9	31.5 ± 4.3	—	29.4 ± 5.0	.844
ESS	13 3. ± 6.8	12 3 ± 3.5	8.2 ± 2.5	5.2 ± 4.5	.01
AHI—events/h	53.3 ± 22.1	58.5 ± 18.1	13.0 ± 5.8	2.9 ± 1.2	<.001
Oxygen desaturation index—number of events/h	50.2 ± 24.3	55.4 ± 21.2	12.7 ± 4.30	1.8 ± 2.1	<.001

Data presented as mean ± SD, unless otherwise stated. OSA: obstructive sleep apnea; CPAP: continuous positive airway pressure; ESS: Epworth Sleepiness Scale; AHI: apnea-hypoapnea index. ^#^Baseline values of the treated patients with OSA refer to the diagnostic time, before starting the treatment with CPAP; ^¶^comparisons between groups were performed by ANOVA or chi-squared test.

## Abbreviations

CPAP: Continuous positive airway pressure
HIF1*α*: Hypoxia-inducible factor 1
HV: Healthy volunteer
IH: Intermittent hypoxia
OSA: Obstructive sleep apnea
VEGF: Vascular endothelial growth factor.

## Supplementary Materials

Supplementary Materials Supplementary Figure 1: obstructive sleep apnea monocytes enhance HIF1*α* and vascular endothelial growth factor mRNA expression. Supplementary Figure 2: the intracellular oxygen concentration in the intermittent hypoxia model. Supplementary Figure 3: images of obstructive sleep apnea monocytes exhibiting tumor-promoting activity.
