# Supplementary material for: Obstructive Sleep Apnea Monocytes Exhibit High Levels of Vascular Endothelial Growth Factor Secretion, Augmenting Tumor Progression
Source: Mediators Inflamm. 2018 May 28;2018:7373921. doi: 10.1155/2018/7373921 (PMC5994578; doi:10.1155/2018/7373921)
Supplement: Supplementary Materials — Supplementary Figure 1: obstructive sleep apnea monocytes enhance HIF1α and vascular endothelial growth factor mRNA expression. Supplementary Figure 2: the intracellular oxygen concentration in the intermittent hypoxia model. Supplementary Figure 3: images of obstructive sleep apnea monocytes exhibiting tumor-promoting activity. [file 7373921.f1.pdf]

Supplementary Data

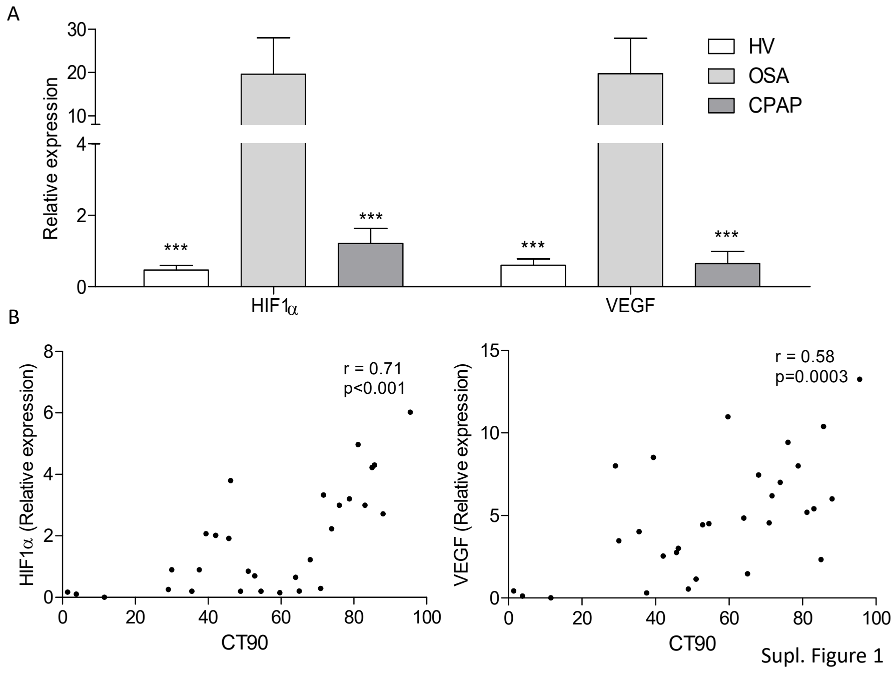

Supplementary Figure 1. Obstructive sleep apnea monocytes enhance HIF1α and vascular endothelial growth factor mRNA expression.

(**A**) CD14$^+$ monocytes were isolated from HV (open bars, n=20 randomly selected), OSA (light gray bars, n=20 randomly selected) and CPAP (dark gray bars, n=20 randomly selected). HIF1α and VEGF mRNA expression by qPCR; ***p<.001, using the Mann-Whitney test. (**B**) The effect of intermittent hypoxia on intracellular oxygen concentration in CD14$^+$ monocytes. Lifetimes of phosphorescent probe are shown. The intracellular oxygen levels were analyzed by the ability of oxygen to quench the excited state of an oxygen-sensitive probe from intracellular oxygen concentration assay (Abcam, ab197245).

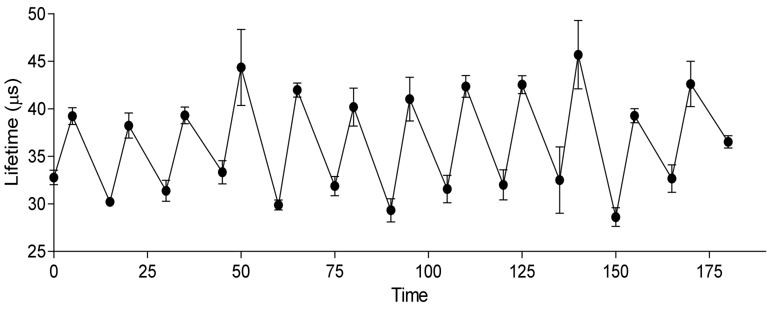

Supl. Figure 2

Supplementary Figure 2. The intracellular oxygen concentration in the intermittent hypoxia model.

The effect of intermittent hypoxia on intracellular oxygen concentration in CD14^{+} monocytes. Lifetimes of phosphorescent probe are shown.

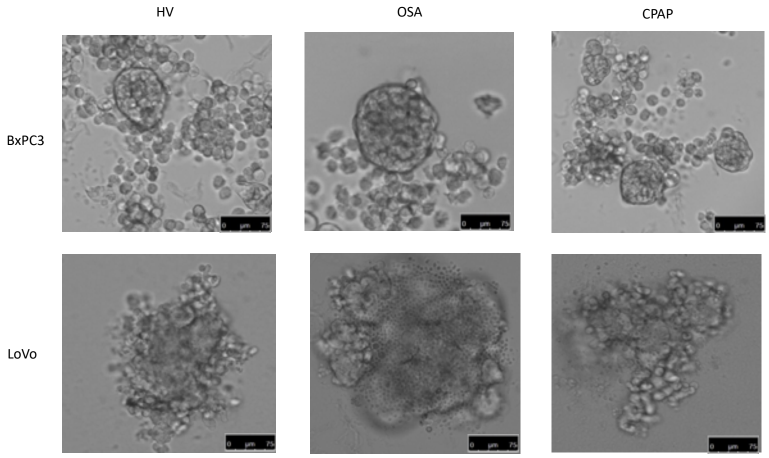

Supl. Figure 3

Supplementary Figure 3. Images of obstructive sleep apnea monocytes exhibiting tumor-promoting activity.

$CD14^+$ monocytes were isolated from HV (n=20 randomly selected), OSA (n=20 randomly selected) and CPAP (n= 20 randomly selected). They were then cocultured in a tumor 3D *in vitro* model using human pancreas (BxPC3) or colon (LoVo) tumor cell lines (3 replicates for each participant). 3D tumor images are shown.
